# Upfront radical surgery with total mesorectal excision followed by adjuvant FOLFOX chemotherapy for locally advanced rectal cancer (TME-FOLFOX): an open-label, multicenter, phase II randomized controlled trial

**DOI:** 10.1186/s13063-020-04266-6

**Published:** 2020-04-07

**Authors:** Jii Bum Lee, Han Sang Kim, Inkyung Jung, Sang Joon Shin, Seung Hoon Beom, Jee Suk Chang, Woong Sub Koom, Tae Il Kim, Hyuk Hur, Byung Soh Min, Nam Kyu Kim, Sohee Park, Seung-Yong Jeong, Jeong-Heum Baek, Seon Hahn Kim, Joon Seok Lim, Kang Young Lee, Joong Bae Ahn

**Affiliations:** 1 Division of Medical Oncology, Department of Internal Medicine, Yonsei Cancer Center, Yonsei University College of Medicine, 50-1 Yonsei-ro, Seodaemun-gu, Seoul, 03722 South Korea; 2 Brain Korea 21 Plus Project for Medical Sciences, Yonsei University College of Medicine, Seoul, South Korea; 3 Division of Biostatistics, Department of Biomedical Systems Informatics, Yonsei University College of Medicine, Seoul, South Korea; 4 Department of Radiation Oncology, Yonsei Cancer Center, Yonsei University College of Medicine, Seoul, South Korea; 5 Department of Internal Medicine and Institute of Gastroenterology, Yonsei University College of Medicine, Seoul, South Korea; 6 Division of Colon and Rectal Surgery, Department of Surgery, Severance Hospital, Yonsei University College of Medicine, Seoul, South Korea; 7 Department of Biostatistics, Graduate School of Public Health, Yonsei University, Seoul, South Korea; 8 Department of Surgery, Seoul National University Hospital, Seoul National University College of Medicine, Seoul, South Korea; 9 Division of Colon and Rectal Surgery, Department of Surgery, Gachon University Gil Medical Center, Gachon University School of Medicine, Incheon, South Korea; 10 Department of Surgery, Korea University Anam Hospital, Seoul, South Korea; 11 Department of Radiology, Severance Hospital, Yonsei University College of Medicine, Seoul, South Korea

**Keywords:** Adjuvant chemotherapy, Clinical trial, FOLFOX, Locally advanced rectal cancer, Total mesorectal excision

## Abstract

**Background:**

Preoperative chemoradiotherapy (PCRT) followed by surgery and adjuvant chemotherapy is the current standard treatment for stage II/III rectal cancer. However, radiotherapy in the pelvic area is commonly associated with complications such as anastomotic leakage, sexual dysfunction, and fecal incontinence. Recently, the MERCURY study showed that preoperative high-resolution magnetic resonance imaging (MRI) helped to selectively avoid PCRT. It remains unclear whether PCRT is necessary in patients who can achieve a negative circumferential resection margin (CRM) with surgery alone and in patients with cT_1–2_N_1_ or cT_3_N_0_ without CRM involvement and lateral lymph node metastasis. This study aims to evaluate the efficacy of upfront radical surgery with total mesorectal excision (TME) followed by adjuvant chemotherapy with folinic acid (or leucovorin), fluorouracil, and oxaliplatin (FOLFOX) versus the current standard treatment in patients with surgically resectable, locally advanced rectal cancer.

**Methods:**

This study, named TME-FOLFOX, is a prospective, open-label, multicenter, phase II randomized trial. Patients with locally advanced rectal cancer will be randomized to receive PCRT followed by TME and adjuvant chemotherapy (arm A) or upfront radical surgery with TME followed by adjuvant FOLFOX chemotherapy (arm B). Clinical stage II/III rectal cancer without CRM involvement and lateral lymph node metastasis will be defined using preoperative MRI. The primary endpoint is 3-year disease-free survival (DFS). Secondary endpoints include 5-year DFS, local recurrence rate, systemic recurrence rate, cost-effectiveness, and overall survival. We hypothesized that our experimental group (arm B) will have a 3-year DFS of 75% and a non-inferiority margin of 15%.

**Discussion:**

Identifying whether patients require PCRT is one of the critical issues in locally advanced rectal cancer. This study aims to elucidate whether PCRT may not be required for all patients with stage II/III rectal cancer, especially for the MRI-based intermediate-risk group (with cT_1–2_N_1_ or cT_3_N_0_) without CRM involvement and lateral lymph node metastasis. If the findings indicate that our proposed treatment, which omits PCRT, is non-inferior to the standard treatment, then patients may avoid unnecessary radiation-related toxicity, have a shorter treatment duration, and save on medical costs.

**Trial registration:**

ClinicalTrials.gov, NCT02167321. Registered on 19 June 2014.

## Introduction

Colorectal cancer (CRC) remains the third most common malignancy and the second leading cause of cancer-related death worldwide [1]. In South Korea, the increasing prevalence of the westernized diet is correlated with a rising incidence of CRC. In 23,271 Korean patients who were diagnosed with CRC in 2018, about 50% of CRC cases were categorized as rectal cancer [2].

Currently, the standard treatment for stage II/III rectal cancer is preoperative chemoradiotherapy (PCRT) followed by surgery and adjuvant chemotherapy [3]. In the past, local relapse after surgery had been the main issue with treatment [4]. However, since the introduction of total mesorectal excision (TME), the incidence of local recurrence has decreased to < 10% [5–7].

The addition of PCRT also led to a decrease in tumor size and local relapse rate [8, 9] and an increase in disease-free survival (DFS); however, it did not have an impact on overall survival (OS) [10]. Thus, the need to control distant metastasis poses as a challenging issue. Although radiotherapy is an effective means of local control, complications such as anastomotic leakage, sexual dysfunction, and fecal incontinence are very common in patients receiving radiotherapy in the pelvic area [11–13]. In addition, PCRT usually takes up to 3 months [14], thereby prolonging the treatment period and increasing the financial burden on patients.

Adjuvant chemotherapy is vital to controlling the systemic recurrence rate, especially in locally advanced rectal cancer. Adjuvant chemotherapy with folinic acid (or leucovorin), 5-fluorouracil (5-FU), and oxaliplatin (FOLFOX) is associated with a 3-year DFS superior to that of 5-FU monotherapy for stage III rectal cancer [15, 16]. The pivotal ADORE trial and the German CAO/ARO/AIO-04 study further supported the efficacy of FOLFOX in the adjuvant setting [17, 18].

Despite the multimodality of the approach to treating locally advanced rectal cancer, some patients are not eligible for PCRT. In the MERCURY study, preoperative high-resolution magnetic resonance imaging (MRI) helped to selectively avoid PCRT [19]. The QuickSilver study also showed that selecting patients with a “good prognosis” using MRI resulted in a low rate of positive circumferential resection margin (CRM) in patients who received upfront surgery [20]. Therefore, the identification of patients who are not eligible for PCRT is one of the critical issues in locally advanced rectal cancer. Whether PCRT is a prerequisite for patients who can achieve a negative CRM with surgery alone warrants further study [21]. If surgery alone can provide local control, then patients can receive adjuvant treatment earlier, preventing systemic relapse.

In this study, we aim to evaluate the efficacy of upfront radical surgery with TME followed by adjuvant FOLFOX chemotherapy in patients with surgically resectable, locally advanced rectal cancer. We hypothesized that the 3-year DFS of patients receiving our proposed treatment will be non-inferior to that of patients receiving the current standard treatment for stage II/III rectal cancer.

## Patients and methods

### Study design

**Fig. 1:**
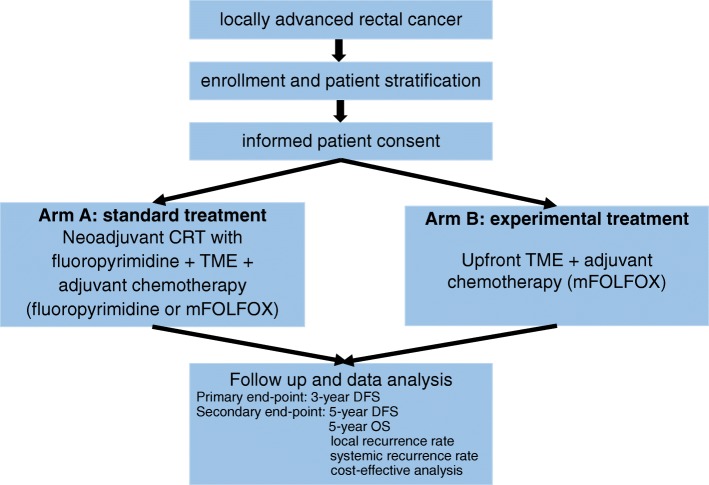
Flow diagram of the trial. DFS disease-free survival, OS overall survival, TME total mesorectal excision, CRT chemoradiotherapy, mFOLFOX modified FOLFOX, folinic acid (or leucovorin), 5-fluorouracil, and oxaliplatin

**Fig. 2:**
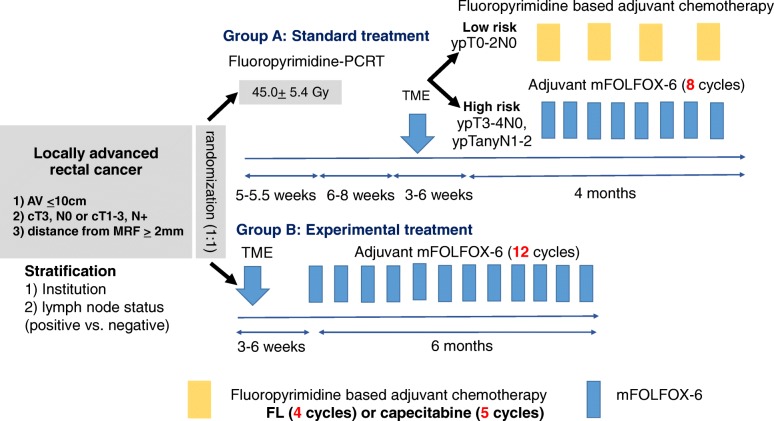
Study treatment. PCRT preoperative chemoradiotherapy, TME total mesorectal excision, AV anal verge, MRF mesorectal fascia, FL 5-fluorouracil and leucovorin, mFOLFOX: modified FOLFOX, folinic acid (or leucovorin), 5-fluorouracil, and oxaliplatin

This study is an open-label, multicenter, phase II randomized controlled trial and will include patients from five tertiary hospitals in South Korea. Patients will be randomly allocated in a 1:1 ratio using random permuted blocks. Factors such as institution and clinical lymph node stage (positive or negative) will be considered when allocating the subjects into one of the following groups: standard treatment group (arm A) or experimental treatment group (arm B) (Fig. 1). Initially, arm A will receive fluoropyrimidine-based or capecitabine-based PCRT (45 ± 5.4 Gy/28 fractions/5.5 weeks, concurrent) followed by TME. Patients will be further stratified into low-risk or high-risk groups for recurrence based on their clinicopathological findings (Fig. 2).

High-risk features include T4 lesion, poor histologic grade, peritumoral lymphovascular involvement, perineural invasions, bowel obstruction at initial presentation, T3 lesion with localized or impending perforation, and an indeterminate or positive margin [22]. Low-risk patients (with ypT_0–2_N_0_) will receive adjuvant chemotherapy with fluoropyrimidine and leucovorin or with capecitabine. High-risk patients (with ypT_3–4_N_0_ or ypT_any_N_1–2_) will be treated with 8 cycles of adjuvant FOLFOX chemotherapy. Arm B will undergo TME followed by 12 cycles of adjuvant FOLFOX chemotherapy.

The regimens to be used in this study are as follows. During radiotherapy, patients may be given 5-FU or capecitabine. A fluoropyrimidine-based regimen of 5-FU 400 mg/m^2^ and leucovorin 20 mg/m^2^ will be given intravenously on days 1–3 and days 29–31. Capecitabine 825 mg/m^2^ will be administered orally twice a day during radiotherapy.

In the adjuvant setting, low-risk patients will receive either the leucovorin regimen or capecitabine. The postoperative leucovorin regimen will consist of 5-FU 400 mg/m^2^ and leucovorin 20 mg/m^2^ given as an intravenous bolus on days 1–5 every cycle (28 days). Capecitabine 1250 mg/m^2^ will be administered orally twice a day on days 1–14 every cycle (21 days). The high-risk group of arm A and arm B will be treated with FOLFOX for 8 and 12 cycles, respectively. The regimen consists of: 5-FU 400 mg/m^2^ administered as an intravenous bolus on day 1, followed by 1200 mg/m^2^ given intravenously over 24 h on days 1–2; oxaliplatin 85 mg/m^2^ administered intravenously; and leucovorin 200 mg/m^2^ administered intravenously on day 1.

Patients are to visit the clinic every 3 months for 3 years after surgery and 6 months thereafter. Follow-up appointments will include physical examination, complete blood count, routine chemistry including liver and kidney function tests, and serum carcinoembryonic antigen test. Chest radiography and abdominal and pelvic computed tomography will be performed every 6 months for 5 years. A routine colonoscopy will also be performed 1, 3, and 5 years after surgery. Every year for 3 years, we will ask patients to answer surveys to evaluate their quality of life.

### Study population

Patient selection is based on the following inclusion and exclusion criteria.

#### Inclusion criteria

1. Histologically confirmed adenocarcinoma of the rectum below 10 cm from the anal verge
2. Locally advanced rectal cancer (T_3_N_0_ or T_1–3_N_positive_)
3. No evidence of para-aortic, common, or external iliac lymph node metastasis
4. Distance of > 2 mm between the primary tumor and mesorectal fascia on pelvic MRI
5. Male or female aged 19–80 years
6. Eastern Cooperative Oncology Group (ECOG) performance status of 0–2
7. Preoperative American Society of Anesthesiologists (ASA) physical status class of I–III
8. No prior systemic chemotherapy including chemotherapy, immunotherapy, or radiotherapy for rectal cancer
9. No previous history of radiotherapy within the pelvic cavity
10. Adequate organ function based on the following parameters:
  - Absolute neutrophil count ≥ 1.5 × 10^3^/L
  - Platelet count ≥ 100 × 10^9^/L
  - Adequate renal function: creatinine ≤ 1.5 times the upper limit of normal (ULN) or glomerular filtration rate of creatinine clearance (calculated using the Cockcroft–Gault formula) > 50 ml/min
  - Adequate hepatic function: alanine aminotransferase-to-aspartate aminotransferase ratio ≤ 2.5 × ULN or total bilirubin ≤ 1.5 × ULN
11. Patients who are willing and able to comply with the protocol during the study
12. Patients with written informed consent

#### Exclusion criteria

1. Rectal malignancy other than adenocarcinoma or adenocarcinoma that developed from inflammatory bowel disease
2. Suspicious distant metastasis
3. Grade ≥ 1 peripheral neuropathy according to the National Cancer Institute Common Terminology Criteria for Adverse Events (CTCAE)
4. Patients receiving concomitant treatment with drugs interacting with 5-FU or oxaliplatin (e.g., flucytosine, phenytoin, warfarin)
5. Uncontrolled and significant cardiovascular disease with heart failure of class III or IV according to the New York Heart Association (NYHA) classification, myocardial infection, or uncontrolled angina pectoris within the past 6 months
6. Prior hypersensitivity reaction to fluoropyrimidine or known dihydropyrimidine dehydrogenase deficiency
7. Hereditary disease such as galactose intolerance, Lapp lactase deficiency, or glucose–galactose malabsorption
8. Known hypersensitivity to platinum-based drugs, leucovorin, or capecitabine
9. Treatment with bevacizumab, cetuximab, oxaliplatin, or irinotecan
10. Uncontrolled active infection or serious concomitant systemic disorders
11. Patients who received organ transplantation requiring immunosuppressive treatment
12. Uncontrolled epilepsy or psychiatric disease
13. A pregnant or lactating female patient

### Safety and quality

Adverse events will be evaluated according to CTCAE version 4.0. Other measures also include total score for function of urination (International Prostate Symptom Score (IPSS)) and defecation score (Wexner’s score). Quality of life will be assessed using the Korean version of the European Organization for Research and Treatment of Cancer Quality-of-Life Questionnaire (EORTC QLQ-C30).

## Results

The primary endpoint is 3-year DFS of patients receiving our proposed treatment versus the standard treatment for surgically resectable, locally advanced rectal cancer. DFS is defined as the time from randomization to disease progression or death from any cause. Secondary endpoints include 5-year DFS, local recurrence rate, systemic recurrence rate, cost-effectiveness, and OS. OS is defined as the time from initial diagnosis to death from any cause.

### Statistical analysis

The primary purpose of this study is to test the non-inferiority of TME followed by 12 cycles of adjuvant FOLFOX chemotherapy to the current standard treatment of PCRT followed by TME and adjuvant chemotherapy. This study is based on the results of the COREAN trial, which showed a 3-year DFS of 72.5% and 79.2% for open and laparoscopic surgery of mid and low rectal cancer, respectively [23]. We estimated that experimental group (arm B) will have a 3-year DFS of 75% and a non-inferiority margin of 15% [24]. To prove non-inferiority [25], we set the upper limit of the one-sided 85% confidence interval of the difference as 15%. With a statistical power of 70%, a one-sided α error of 15%, and a 10% dropout rate, we estimated a total of 90 patients for this study, with 45 patients for each arm.

Our analysis will be based on an intention-to-treat population and a per-protocol population. Continuous and categorical variables will be analyzed using the Mann–Whitney test and Fisher’s exact test, respectively. Spearman’s correlation test will be used for correlation analyses. The Kaplan–Meier method will be used to estimate DFS, OS, and local and distant recurrence, and the log-rank (Mantel–Cox) tests to compare survival distribution. The significance level is set at *p* < 0.05, and all statistical tests will be two-sided. There will be no interim analysis.

### Translational analyses

Using next-generation sequencing technology, we will perform biomarker analysis and gene expression profiling of surgically resected rectal specimens. The results may provide insight into biomarkers that can predict responses, outcomes, and recurrence in patients with locally advanced rectal cancer.

### Data collection, management, and monitoring

Patient data will be effectively managed using the eVelos system, a web-based clinical trial management system (http://kcpc.ncc.re.kr; Velos Inc., Fremont, CA, USA). We estimated the patient accrual and study duration to take 4 and 5 years, respectively.

## Discussion

Although the multimodality of approach for treating locally advanced rectal cancer has lowered the local recurrence rate and improved DFS, approximately one-third of patients still experience systemic recurrence [10]. PCRT may help to downsize tumors and provide local control [23], but its role is questionable if patients with a negative CRM may be eligible for TME alone using high-resolution MRI [19]. Our study aims at proving the non-inferiority of TME followed by 12 cycles of adjuvant FOLFOX chemotherapy to the standard treatment of PCRT followed by TME and adjuvant chemotherapy. If our findings reveal that our proposed treatment method, which omits PCRT, is non-inferior to the standard treatment, then patients may avoid unnecessary radiation-related toxicity, have shorter treatment duration, and save on medical costs. The results of our translational research also provide important insights into biomarkers associated with radiotherapy.

Selective approaches to radiotherapy for clinical stage II/III rectal cancer are currently one of the unmet needs of patients in this field. Our study will be comparable to the MERCURY, OCUM, or QuickSilver study, all of which used high-resolution MRI to select patients with a good prognosis, defined as a negative CRM [19, 20, 26]. These studies had CRM rates of 2–5% and 5-year local recurrences rates of 2.0–3.3% in patients without PCRT, suggesting that precise clinical staging based on high-resolution MRI can help avoid unnecessary PCRT in patients with a good prognosis. Considering the impact of PCRT on local control, the rate of CRM involvement and the rate of completing a TME procedure will be critical factors in this study.

Our study was registered at ClinicalTrials.gov (NCT02167321) on June 19, 2014. Patient accrual began in December 2014. Patients will be followed up until June 2024.

## Data Availability

The datasets used or analyzed in this trial may be available upon reasonable request to the corresponding author.

## Abbreviations

ASA: American Society of Anesthesiologists
CRC: Colorectal cancer
CRM: Circumferential resection margin
CTCAE: National Cancer Institute Common Terminology Criteria for Adverse Events
DFS: Disease-free survival
ECOG: Eastern Cooperative Oncology Group
EORTC QLQ-C30: Korean version of the European Organization for Research and Treatment of Cancer Quality-of-Life Questionnaire
FOLFOX: Folinic acid (or leucovorin), 5-fluorouracil, and oxaliplatin
5-FU: 5-Fluorouracil
IPSS: International Prostate Symptom Score
MRI: Magnetic resonance imaging
NYHA: New York Heart Association
OS: Overall survival
PRCT: Preoperative chemoradiotherapy
TME: Total mesorectal excision
ULN: Upper limit of normal
